# Comparative performance of cobas 4800 HPV Test and Anyplex II HPV HR for high-risk human papillomavirus detection

**DOI:** 10.1128/jcm.00200-25

**Published:** 2025-07-09

**Authors:** Luani R. Godoy, Mariam El-Zein, Elizaveta Padalko, Bo Verberckmoes, Bodine Van Eenooghe, Heleen Vermandere, Sónia Dias, Ana Gama, Bernardo Vega Crespo, Vivian Alejandra Neira, Eduardo L. Franco, Adhemar Longatto-Filho, for the ELEVATE Study Group, Yasmin M. Guimarães

**Affiliations:** 1 Division of Cancer Epidemiology, McGill University 645355, Montreal, Québec, Canada; 2 Molecular Oncology Research Center, Barretos Cancer Hospital 67766 , Barretos, São Paulo, Brazil; 3 Department of Diagnostic Sciences, Faculty of Medicine and Health Sciences, Ghent University 54498 , Ghent, Belgium; 4 International Centre for Reproductive Health, Department of Public Health and Primary Care, Faculty of Medicine and Health Sciences, Ghent University 129177 , Ghent, Belgium; 5 NOVA National School of Public Health, Public Health Research Centre, Comprehensive Health Research Center (CHRC), LA-REAL, NOVA University Lisbon 449872 , Lisbon, Portugal; 6 Facultad de Ciencias Médicas, Universidad de Cuenca 27889, Cuenca, Ecuador; 7 Department of Biosciences, Faculty of Chemical Sciences, University of Cuenca 27889, Cuenca, Ecuador; 8 Life and Health Sciences Research Institute (ICVS), University of Minho 467460 , Braga, Portugal; 9 ICVS/3B’s—PT Government Associate Laboratory, Braga, Portugal; 10 Laboratory of Medical Investigation (LIM14), Faculty of Medicine, São Paulo State University 67785 , São Paulo, Brazil; St Jude Children's Research Hospital, Memphis, Tennessee, USA

**Keywords:** human papillomavirus, cobas 4800 HPV test, Anyplex II HPV HR assay, performance

## Abstract

Numerous molecular tests are available to detect human papillomavirus (HPV). We compared the analytical performance of cobas and Anyplex for detection of high-risk (HR) carcinogenic HPV genotypes, assessed the composition of HPV types (other than 16 and 18) that influenced cobas performance, and considered the impact of viral load on test performance. We used data from the Early Detection of Cervical Cancer in Hard-to-Reach Populations of Women Through Portable and Point-of-Care HPV Testing project, which involved collection (2019–2022) of cervicovaginal samples from 1,042 women aged 21–74 years in Belgium (*n* = 244), Portugal (*n* = 309), Brazil (*n* = 244), and Ecuador (*n* = 245). Samples were tested by cobas (provides individual results for HPV16 and HPV18 and a pooled result for 12 other HR-HPV types) and Anyplex (provides separate results for 14 HR-HPVs). We calculated HPV positivity by each test and compared performance between tests by calculating Cohen’s kappa statistics. Based on 938 samples with complete data from both tests, positivity rates by cobas were 13.4%, 3.6%, 34.3%, and 45.3% for HPV16, HPV18, 12 pooled HR-HPVs, and any HR-HPV, respectively. Corresponding HPV positivity rates by Anyplex were 14.9%, 3.7%, 37.9%, and 50.0% for the same categories, respectively, with high concordance; kappa statistics were 0.90, 0.87, 0.82, and 0.85, respectively. Based on 355 samples that tested positive for at least 1 of the 12 pooled HR-HPVs, most types showed high agreement (80.9%–100.0%) between individual-Anyplex and pooled-cobas HPV results, except for HPV68 (61.3% agreement). Our findings suggest that the two commercial tests may have different performances, depending on the specific HPV types detected, emphasizing the need for continued research on conditions that may affect these tests, especially for less common or less studied HPV types.

**IMPORTANCE:**

This study compared two commercial tests—cobas and Anyplex—for detecting high-risk HPV types in women undergoing routine cervical cancer screening or referred for colposcopy. Both tests provide separate results for HPV16 and HPV18, but Anyplex also identifies the remaining 12 high-risk HPV types individually, while cobas groups them together. Overall, we found a high level of agreement between the two tests, supporting their use in clinical practice. However, differences in detecting certain HPV types, particularly those that are less common or less studied, emphasize the importance of choosing the right test. As more countries switch to HPV-based cervical cancer screening, using tests that provide detailed results could help improve risk assessment and optimize patient care.

## INTRODUCTION

Human papillomavirus (HPV) is the most common sexually transmitted infection, with over 200 known genotypes recognized based on DNA sequence data (1). Although most cervical HPV infections resolve spontaneously, persistent infection with high-risk (HR) genotypes (particularly HPVs 16 and 18) may cause premalignant lesions, which, if left untreated, could eventually lead to the development of invasive cervical cancer (2). The primary screening tool for cervical cancer has dramatically shifted in many countries from the traditional gynecological Pap smear cytology-based screening for the presence of precancerous cell changes in the cervix to molecular HPV testing of nucleic acids of the virus (3). This paradigm shift was motivated by the clinical superiority of HPV testing (4, 5) for detecting high-risk HPV types associated with cervical carcinogenesis (6). In addition, the World Health Organization’s global strategy (7) to eliminate cervical cancer by reducing the age-standardized incidence to less than 4 per 100,000 by the end of the 21st century is premised upon the implementation of HPV testing as the primary cervical cancer screening modality. Most management algorithms prioritize the detection of HPV16/18 for immediate referral to colposcopy. Extended genotyping beyond these two types has not yet been integrated into routine triage practices. Its primary potential lies in enhancing future risk stratification strategies and supporting epidemiological surveillance efforts.

Although more than 250 molecular HPV tests are available (8), only 8 have been validated (against Hybrid Capture 2 HPV DNA Test [Qiagen, Maryland, USA] or GP5+/6+ PCR-enzyme immunoassay [EIA]) based on international criteria and have shown inter- and intralaboratory reproducibility for HPV-based primary cervical cancer screening (9). These include Abbott RealTime High Risk HPV Test (Abbot, Wiesbaden, Germany), cobas 4800 HPV Test (Roche, Pleasanton, CF, USA), Anyplex II HPV HR Detection (Seegene, Seoul, South Korea), BD Onclarity HPV Assay (Becton Dickinson, Sparks, MD, USA), HPV-Risk Assay (Self-Screen BV, Amsterdam, The Netherlands), PapilloCheck HPV-Screening Test (Greiner Bio-One, Frickenhausen, Germany), Xpert HPV (Cepheid, Sunnyvale, CA, USA), and Alinity m HR HPV Assay (Abbot). Although these assays show similar overall performance in detecting CIN2+, they differ in key design aspects, such as target regions, analytical sensitivity, and genotyping strategies. These differences can result in variations in the specific HPV infections detected, consequently influencing clinical sensitivity and specificity (10).

The cobas 4800 HPV test (cobas for short henceforward) and Anyplex II HPV HR detection assay (Anyplex for short henceforward) are two well-established commercial HPV tests that differ in their genotyping abilities. cobas targets the HPV L1 (viral capsid) region, concurrently offering partial genotyping by detecting HPV16 and HPV18 individually and a pooled test result for the 12 other HR-HPV genotypes (HPVs 31, 33, 35, 39, 45, 51, 52, 56, 58, 59, 66, and 68). Anyplex individually detects the above 14 HR-HPVs in a single reaction; however, the specific genomic targets are not publicly disclosed by the manufacturer and may encompass regions beyond L1.

Since many countries are in the process of transitioning to HPV primary screening, selecting the most appropriate HPV test is a critical component of planning an HPV test-based cervical cancer screening program. Incorporating genotype-specific identification of the abovementioned 14 HPVs, recognized by the International Agency for Research on Cancer to be the most clinically relevant genotypes (11), could potentially improve patient care management. Concordance between cobas and Anyplex for the detection of HR-HPVs is thus important when considering implementation options in routine clinical practice and risk-based triage of HR-HPV screen-positive women.

We evaluated and compared the performance of cobas and Anyplex for the detection of HR-HPV types. Then, we assessed the composition of HPV types (other than HPVs 16 and 18) that influenced the performance of cobas, a test approved in 2014 by the US Food and Drug Administration and widely used in the USA. We also assessed the impact of HPV viral load on test performance.

## MATERIALS AND METHODS

### Study design and procedures

Participating subjects were part of the **E**ar**l**y D**e**tection of Cer**v**ical C**a**ncer in Hard-to-Reach Populations of Women Through Portable and Point-of-Care HPV **Te**sting (ELEVATE) cross-sectional study carried out among women attending either (i) routine screening for cervical cancer or (ii) colposcopy following referral. Details of the ELEVATE study design and data collection procedures were previously reported (12). Briefly, the study was conducted in Belgium (University Hospital of Ghent), Portugal (Instituto Português de Oncologia de Lisboa Francisco Gentil and several primary healthcare units), Brazil (Barretos Cancer Hospital), and Ecuador (Sociedad de Lucha Contra el Cáncer). Eligible women included those aged 21–74 years, who were sexually active and not pregnant, had no history of hysterectomy, and had never been treated for cervical lesions or cancer. We excluded women who were menstruating at the time of their visit or had penetrative vaginal sex within the last 48 hours before their visit. Cytology was performed at the routine screening visit, and/or cervical biopsies were taken from visible lesions at the colposcopy visit following best clinical practices. Interpretation of cytology results and histological assessment were, respectively, done by local cytotechnicians and pathologists.

### Data collection and procedures

An interviewer-administered questionnaire was used to collect data on sociodemographic characteristics; answers were transcribed into the web-based application Research Electronic Data Capture. Two cervicovaginal samples were collected from each woman by a gynecologist or nurse. To mimic self-sample collection, the healthcare professional simultaneously inserted two Viba-Brush self-testing devices (Rovers, Oss, The Netherlands) at the same time. In Belgium, Portugal, and Brazil, the brushes were suspended in 20 mL of ThinPrep Pap test vial containing PreservCyt liquid cytology medium (Hologic Inc., Mississauga, ON, Canada), whereas in Ecuador, they were rinsed in Roche cell collection medium (Roche Diagnostics, Indianapolis, IN, USA). After collection, the two cervicovaginal samples were stored at 2^o^C–8°C pending transfer; one was sent to Brazil for HPV genotyping by cobas, and the other was sent to Belgium for HPV genotyping by Anyplex.

### HPV genotyping and viral load

Samples were first tested with cobas (October 2020–September 2022, performed at the Barretos Cancer Hospital using 1 mL of aliquoted sample) and then with Anyplex (June–August 2023, performed at Ghent University Hospital using 300 µL of the aliquoted sample), according to the respective manufacturer’s instructions. Technicians were unaware of the subjects’ previous HPV test results and clinical data. Both tests are automated multiplex real-time PCR-based assays that amplify the human β-globin gene as a built-in internal control for sample adequacy. In case of positivity, cobas provides the cycle threshold (Ct) values that represent relative, rather than absolute, measures of viral load. Ct values are inversely correlated with viral load: low Ct values correspond to high amounts of viral DNA. A sample was considered HPV positive when Ct values for HPV16 were ≤40.50, and those for HPV18 and pooled HPVs were ≤40.00. Anyplex provides semiquantitative viral load level of amplification, which can be measured repeatedly at 30, 40, and 50 cycles during the PCR process. HPV viral load was categorized as high, medium, or low if the Ct value was <31, between 31 and 40, or ≥41, respectively.

### Statistical analysis

Disease endpoints were defined based on a combination of available histology, cytology, and colposcopy data; we ascertained cervical lesion status based on biopsy results and, if not available, on the worst diagnosis between cytology and colposcopy. We summarized, by country, the characteristics of the study population and prevalence of HPV positivity as measured by each assay.

We assessed, for the entire study population and by cervical lesion status, any HPV (defined as having 1 or more of the 14 HR types tested) and genotype-specific concordance between the two assays using Cohen’s kappa statistic. The range of kappa values indicates the extent of agreement: 0–0.2, poor; 0.21–0.4, fair; 0.41–0.6, moderate; 0.61–0.8, good; and 0.81–1.0, excellent (13). We cross-tabulated positivity to the individual HPV genotypes by Anyplex and the cobas positive results for the aggregated genotypes, overall and by country, also considering single (i.e., samples with only one genotype detected) and multiple (i.e., samples with more than one genotype detected) type infections.

To test the hypothesis that viral load could affect test performance, we summarized for HPV16, HPV18, and the 12 pooled HPVs the distribution of Ct values for cobas according to Anyplex test results; the Mann–Whitney test was used to compare the means of Ct values. Likewise, we summarized for each of the 14 HPVs the distribution of viral load for Anyplex according to HPV test results by cobas; the Cochran–Armitage test was used to assess the trend in Anyplex viral load. Statistical analyses were performed in Stata v.18.0 (StatCorp LP, College Station, TX, USA). A *P* value of <0.05 was considered statistically significant.

## RESULTS

**Fig 1:**
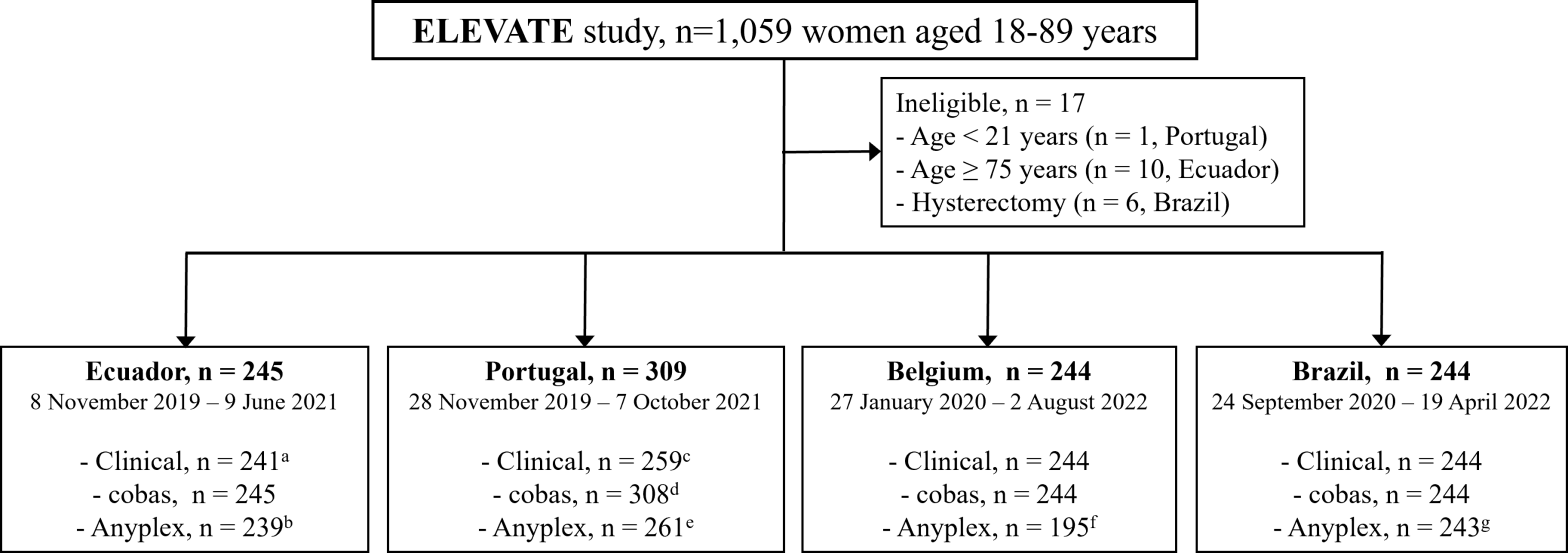
Overview of the study population (*n* = 1042) and collected data by country. Clinical data included biopsy, cytology, and colposcopy results, which, based on the available data, were used to ascertain cervical lesion status as being normal, low-grade, high-grade, and cancer. Refer to Table 1 for further details. ^a^Four samples were inadequate. ^b^Not performed for six samples. ^c^Fourteen samples had inadequate cytology results, and no samples were collected for 37 women. ^d^One sample had an invalid result. ^e^Not performed for 48 samples. ^f^Not performed for 49 samples. ^g^Not performed for one sample. ELEVATE, Early Detection of Cervical Cancer in Hard-To-Reach Populations of Women Through Portable and Point-of-Care HPV Testing.

Figure 1 shows a flowchart of recruitment by country and available data collected between November 2019 and August 2022. A total of 1,042 participants were enrolled, and 938 samples were included in the comparison between cobas and Anyplex. Table 1 summarizes the characteristics of the study population. Overall, the median age of the participants was 43.9 years (interquartile range: 15 years, range 21.6–74.3), and almost half had a secondary or university education (54.9%) and were married or in a relationship (49%). Most samples were classified as normal (*n* = 631, 63.9%), whereas 177 (17.9%), 131 (13.3%), and 49 (5.0%) samples were from women with low-grade cervical lesions, high-grade cervical lesions, and cervical cancer, respectively. The proportion of high-grade cervical lesions and cancer was lowest in Portugal (6.6%) and relatively comparable across Ecuador (20.3%), Belgium (22.9%), and Brazil (23.8%).

**TABLE 1:** Characteristics of the study population (*n* = 1,042), by country^*a*^

Variables	Ecuador *n* = 245	Portugal *n* = 309	Belgium *n* = 244	Brazil *n* = 244
Age (years)				
Mean ± SD	46.9 ± 11.0	42.0 ± 10.2	45.0 ± 9.8	45.8 ± 8.7
Median (IQR)	45.8 (16.7)	41.1 (14.3)	44.6 (16.1)	45.3 (12.8)
Range	29.1–74.3	21.6–72.0	26.5–68.4	24.3–65.0
Education, *n* (%)				
Pre-elementary	37 (15.1)	10 (3.2)	5 (2.0)	47 (19.3)
Primary	98 (40.0)	31 (10.0)	7 (2.9)	47 (19.3)
Secondary	62 (25.3)	42 (13.6)	58 (23.8)	108 (44.3)
University	48 (19.6)	60 (19.4)	152 (62.3)	42 (17.2)
Missing	0	166 (53.7)	22 (9.0)	0
Marital status, *n* (%)				
Single	39 (15.9)	40 (13.0)	37 (15.2)	65 (26.6)
In a relationship/married	158 (64.5)	74 (24.0)	162 (66.4)	117 (48.0)
Divorced	29 (11.8)	26 (8.4)	21 (8.6)	49 (20.1)
Widow	19 (7.8)	2 (0.6)	2 (0.8)	13 (5.3)
Missing	0	167 (54.0)	22 (9.0)	0
Region of birth, *n* (%)				
South America/Caribbean	245 (100)	28 (9.1)	2 (0.8)	244 (100)
Europe	0	85 (27.5)	211 (86.5)	0
Africa	0	12 (3.9)	4 (1.6)	0
Asia	0	15 (4.8)	6 (2.4)	0
Missing	0	169 (54.7)	21 (8.6)	0
Ethnicity, *n* (%)				
White	9 (3.7)	--	209 (85.7)	109 (44.7)
Brown	230 (93.9)	--	3 (1.2)	105 (43.0)
Indigenous	4 (1.6)	--	0	2 (0.8)
Black	2 (0.8)	--	3 (1.2)	20 (8.2)
Asian	0	--	5 (2.0)	7 (2.9)
Missing	0	309 (100)	24 (9.9)	1 (0.4)
Cervical lesion status,^*b*^ *n* (%)				
Normal	147 (61.0)	187 (72.2)	144 (59.0)	153 (62.7)
Low-grade cervical lesion	45 (18.7)	55 (21.2)	44 (18.0)	33 (13.5)
High-grade cervical lesion	19 (7.9)	16 (6.2)	44 (18.0)	52 (21.3)
Cervical cancer	30 (12.4)	1 (0.4)	12 (4.9)	6 (2.5)
Missing	4	50	0	0

^*a*^ IQR, interquartile range; SD, standard deviation; --, data not collected.

^*b*^ Ascertained based on biopsy results (*n* = 257), when available; otherwise, the worst diagnosis between cytology (*n* = 680) and colposcopy (*n* = 51) results was used. **Normal** includes biopsy-confirmed normal (*n* = 70), cytology-interpreted negative for intraepithelial lesion or malignancy (*n* = 519), and no lesional tissue on colposcopy (*n* = 42). **Low-grade** includes biopsy-confirmed cervical intraepithelial neoplasia grade 1 (*n* = 53), cytology-interpreted low-grade squamous intraepithelial lesion (*n* = 84), atypical squamous cells of undetermined significance (*n* = 31), and low-grade lesion on colposcopy (*n* = 9). **High-grade** includes biopsy-confirmed CIN2 (*n* = 41) and CIN3 (*n* = 46), as well as cytology-interpreted high-grade squamous intraepithelial lesion (*n* = 22), atypical squamous cells, cannot exclude a high-grade squamous intraepithelial lesion (*n* = 21), and atypical glandular cells of undetermined significance (*n* = 1). **Cancer** includes biopsy-confirmed squamous cell carcinoma (SCC, *n* = 29), adenocarcinoma (*n* = 11), and cervical cancer (no distinction between SCC and adenocarcinoma, *n* = 7), as well as cytology-interpreted cancer (SCC, *n* = 1, and adenocarcinoma, *n* = 1). Proportions reported are based on valid samples, excluding missing data.

Table 2 details the paired results of cobas and Anyplex for HPV detection, separately for each country. Results are grouped with an emphasis on individual HPV16 and HPV18 positivity, with the remainder of 12 HR types pooled together. Overall, for samples tested by cobas, 139 (13.4%) were HPV16^+^; 37 (3.6%) were HPV18^+^; 357 (34.3%) were positive for the 12 other HPV types; and 472 (45.3%) were positive for any HPV type. Equivalent values, when samples were tested by Anyplex, were 140 (14.9%), 35 (3.7%), 355 (37.9%), and 469 (50.0%). HPV16 prevalence varied by country (Ecuador: 13.5% by cobas and 13.8% by Anyplex, Portugal: 19.5% by cobas and 24.9% by Anyplex, Belgium: 7.4% by cobas and 6.7% by Anyplex, and Brazil: 11.5% by cobas and 11.9% by Anyplex). Differences in positivity for any HPV type were also observed across countries.

**TABLE 2:** HPV test results (*n* [%]) by cobas (*n* = 1,041) and Anyplex (*n* = 938), by country^*a*^

Detection of HR-HPV types			Ecuador		Portugal		Belgium		Brazil	
HPV16	HPV18	12 pooled HPVs^*b*^	cobas *n* = 245	Anyplex^*c*^ *n* = 239	cobas^*d*^ *n* = 308	Anyplex^*e*^ *n* = 261	cobas *n* = 244	Anyplex^*f*^ *n* = 195	cobas *n* = 244	Anyplex^*g*^ *n* = 243
+	−	−	26 (10.6)	25 (10.5)	40 (13.0)	43 (16.5)	10 (4.1)	8 (4.1)	17 (7.0)	18 (7.4)
−	+	−	6 (2.4)	5 (2.1)	6 (1.9)	6 (2.3)	5 (2.0)	4 (2.0)	3 (1.2)	3 (1.2)
−	−	+	50 (20.4)	53 (22.2)	111 (36.0)	112 (42.9)	61 (25.0)	52 (26.7)	80 (32.8)	82 (33.7)
+	−	+	7 (2.9)	8 (3.3)	18 (5.8)	20 (7.7)	7 (2.9)	5 (2.6)	8 (3.3)	8 (3.3)
−	+	+	2 (0.8)	2 (0.8)	4 (1.3)	5 (1.9)	2 (0.8)	2 (1.0)	3 (1.2)	3 (1.2)
+	+	−	0	0	0	0	0	0	2 (0.8)	2 (0.8)
+	+	+	0	0	2 (0.6)	2 (0.8)	1 (0.4)	0	1 (0.4)	1 (0.4)
−	−	−	154 (62.9)	146 (61.1)	127 (41.2)	73 (28.0)	158 (64.8)	124 (63.6)	130 (53.3)	126 (51.9)

^*a*^ HPV, human papillomavirus; HR, high risk.

^*b*^ Includes HPVs 31, 33, 35, 39, 45, 51, 52, 56, 58, 59, 66, and 68.

^*c*^ Not performed for six samples.

^*d*^ One sample had an invalid result.

^*e*^ Not performed for 48 samples.

^*f*^ Not performed for 49 samples.

^*g*^ Not performed for one sample.

Overall, based on 938 samples with complete data from both tests, excellent agreement was observed between cobas and Anyplex by lesion category (Table 3). Concordance for any HPV, HPV16, HPV18, and 12 pooled HPVs was 92.4% (kappa = 0.85), 97.5% (kappa = 0.90), 99.0% (kappa = 0.87), and 91.8% (kappa = 0.82), respectively. Discordant results between the two assays were observed for any HPV in 71 samples (47 positive only by Anyplex and 24 positive only by cobas), for HPV16 in 23 samples (15 positive only by Anyplex and eight positive only by cobas), for HPV18 in 9 samples (five positive only by Anyplex and four positive only by cobas), and for 12 pooled HPVs in 77 samples (49 positive only by Anyplex and 28 positive only by cobas). As expected, we observed a positive trend for HPV positivity according to the severity of the underlying cervical lesion status. Concordance was also high between the two tests irrespective of cervical lesion status, ranging from 86.0% (for 12 pooled HPVs, kappa = 0.71) to 100% (for HPV18, kappa = 1.00) in cancer cases.

**TABLE 3:** Concordance of HPV test results between Anyplex and cobas, overall (*n* = 938^*b*^) and by cervical lesion status (*n* = 924^*b*^^,^^*c*^)^*a*^

HR- HPV	Anyplex	Overall			Normal, *n* = 588			Low grade, *n* = 172			High grade, *n* = 121			Cancer, *n* = 43		
		cobas, *n* (%)		Kappa	cobas, *n* (%)		Kappa	cobas, *n* (%)		Kappa	cobas, *n* (%)		Kappa	cobas, *n* (%)		Kappa
		+	−		+	−		+	−		+	−		+	−	
Any HPV^*d*^	+	422(45.0)	47(5.0)	0.8486	176(29.9)	31(5.3)	0.8075	108(62.8)	4( 2.3)	0.9238	98(81.0)	5(4.1)	0.7863	35(81.4)	4(9.3)	0.6195
	−	24(2.6)	445(47.4)		20(3.4)	361(61.4)		2 (1.2)	58 (33.7)		2 (1.7)	16 (13.2)		0(0.0)	4(9.3)	
HPV16	+	125(13.3)	15(1.6)	0.9014	43 (7.3)	8 (1.4)	0.8470	32 (18.6)	2 (1.2)	0.9444	31 (25.6)	2 (1.7)	0.9369	17 (39.5)	3(7.0)	0.8584
	−	8(0.9)	790(84.2)		6 (1.0)	531(90.3)		1 (0.6)	137(79.7)		1(0.8)	87(71.9)		0(0.0)	23(53.5)	
HPV18	+	30(3.2)	5(0.5)	0.8646	9 (1.5)	3 (0.5)	0.7448	8 (4.7)	1 (0.6)	0.8828	6 (5.0)	1 (0.8)	0.9187	7 (16.3)	0(0.0)	1.0000
	−	4(0.4)	899(95.8)		3 (0.5)	573(97.4)		1 (0.6)	162(94.2)		0 (0.0)	114(94.2)		0(0.0)	36 (83.7)	
12 pooled HPVs^*e*^	+	306(32.6)	49(5.2)	0.8235	135(23.0)	33 (5.6)	0.7701	88 (51.2)	4 (2.3)	0.9300	66 (54.5)	5 (4.1)	0.8818	14 (32.6)	3(7.0)	0.7081
	−	28(3.0)	555(59.2)		21 (3.6)	399(67.9)		2 (1.2)	78 (45.3)		2(1.7)	48 (39.7)		3(7.0)	23(53.5)	

^*a*^ HPV, human papillomavirus; HR, high risk.

^*b*^ Includes only samples that had an HPV result by both tests.

^*c*^ Fourteen samples had no clinical results. Cervical lesion status was ascertained based on biopsy results (*n* = 227), when available; otherwise, the worst diagnosis between cytology (*n* = 656) and colposcopy (*n* = 41) results was used. **Normal** includes biopsy-confirmed normal (*n* = 59), cytology-interpreted negative for intraepithelial lesion or malignancy (*n* = 496), and no lesional tissue on colposcopy (*n* = 33). **Low-grade** includes biopsy-confirmed cervical intraepithelial neoplasia grade 1 (*n* = 49), cytology-interpreted low-grade squamous intraepithelial lesion (*n* = 84), atypical squamous cells of undetermined significance (*n* = 31), and low-grade lesion on colposcopy (*n* = 8). **High-grade** includes biopsy-confirmed CIN2 (*n* = 38) and CIN3 (*n* = 40), as well as cytology-interpreted high-grade squamous intraepithelial lesion (*n* = 21), atypical squamous cells, cannot exclude a high-grade squamous intraepithelial lesion (*n* = 21), and atypical glandular cells of undetermined significance (*n* = 1). **Cancer** includes biopsy-confirmed squamous cell carcinoma (SCC, *n* = 25), adenocarcinoma (*n* = 10), and cervical cancer (no distinction between SCC and adenocarcinoma, *n* = 6), as well as cytology-interpreted cancer (SCC, *n* = 1, and adenocarcinoma, *n* = 1).

^*d*^ Positivity to any of HPVs 16, 18, 31, 33, 35, 39, 45, 51, 52, 56, 58, 59, 66, and/or 68.

^*e*^ Includes HPVs 31, 33, 35, 39, 45, 51, 52, 56, 58, 59, 66, and 68.

Based on 355 samples that tested positive by Anyplex for at least 1 of the 12 HPVs (Table 4), high agreement (80.9%-100%) was observed between individual-Anyplex and pooled-cobas HPV results, except for HPV68 (61.3% agreement). Of the 31 samples that tested positive for HPV68 by Anyplex, 12 (38.7%) were negative by cobas for the 12 pooled HPVs. Of the latter, 11 samples were considered “single infection” with HPV68, and one sample was considered to have “multiple infections” with HPVs 18 and 68 (Table S1).

**TABLE 4:** Cross tabulation (*n* [%]) between positivity to individual HPV types by Anyplex and pooled HPV test results by cobas for the 12 other HR-HPVs, overall and by country^*a*^^,^^*b*^

HPV positivity by Anyplex	Overall, *n* = 355		Ecuador, *n* = 63		Portugal, *n* = 139		Belgium, *n* = 59		Brazil, *n* = 94	
	cobas+	cobas−	cobas+	cobas−	cobas+	cobas−	cobas+	cobas−	cobas+	cobas−
HPV31	68 (93.2)	5 (6.8)	9 (100)	0	29 (90.6)	3 (9.4)	15 (88.2)	2 (11.8)	15 (100)	0
HPV33	23 (88.5)	3 (11.5)	2 (100)	0	11 (91.7)	1 (8.3)	5 (83.3)	1 (16.7)	5 (83.3)	1 (16.7)
HPV35	36 (100)	0	2 (100)	0	13 (100)	0	7 (100)	0	14 (100)	0
HPV39	20 (87.0)	3 (13.0)	5 (83.3)	1 (16.7)	6 (85.7)	1 (14.3)	5 (83.3)	1 (16.7)	4 (100)	0
HPV45	29 (90.6)	3 (9.4)	5 (100)	0	13 (92.9)	1 (7.1)	6 (100)	0	5 (71.4)	2 (28.6)
HPV51	34 (80.9)	8 (19.1)	4 (80.0)	1 (20.0)	20 (80.0)	5 (20.0)	4 (66.7)	2 (33.3)	6 (100)	0
HPV52	43 (91.5)	4 (8.5)	11 (91.7)	1 (8.3)	21 (95.4)	1 (4.6)	4 (100)	0	7 (77.8)	2 (22.2)
HPV56	29 (87.9)	4 (12.1)	2 (100)	0	11 (73.3)	4 (26.7)	6 (100)	0	10 (100)	0
HPV58	44 (89.8)	5 (10.2)	9 (90.0)	1 (10.0)	18 (90.0)	2 (10.0)	4 (80)	1 (20)	13 (92.9)	1 (7.1)
HPV59	40 (90.9)	4 (9.1)	11 (91.7)	1 (8.3)	10 (100)	0	6 (66.7)	3 (33.3)	13 (100)	0
HPV66	44 (91.7)	4 (8.3)	4 (66.7)	2 (33.3)	15 (100)	0	11 (100)	0	14 (87.5)	2 (12.5)
HPV68	19 (61.3)	12 (38.7)	2 (40.0)	3 (60.0)	6 (50.0)	6 (50.0)	2 (100)	0	9 (75)	3 (25)

^*a*^ HPV, human papillomavirus; HR, high risk.

^*b*^ Includes only samples that had an HPV result by both tests and are positive by Anyplex for at least one HR-HPV type other than HPVs 16 and 18.

As shown in Table 5, for HPV positive samples by cobas (*n* = 501), the median Ct values were significantly higher in Anyplex HPV-negative than HPV-positive samples (HPV16: 37.8 vs 29.2, *P* value = 0.0002; HPV18: 37.4 vs 31.2, *P* value = 0.0059; 12 pooled HPVs: 37.6 vs 30.1, *P* value = 0.0000). Likewise, in HPV16- and HPV18-positive samples by Anyplex, we found a significant dose–response pattern by viral load tertiles between HPV16 positive and negative samples by cobas (Table 6). A similar trend was found when considering individual-Anyplex and pooled-cobas HPV results, except for HPVs 52, 56, 58, 59, 66, and 68. Specifically for HPV68, the proportion of samples that were cobas negative and had low, moderate, and high viral loads by Anyplex was 35.7%, 42.9% and 33.3%, respectively.

**TABLE 5:** Distribution of Ct values for cobas according to Anyplex test results^*a*^^,^^*b*^

HR-HPV positivity by cobas	HPV test results by Anyplex	Ct values, cobas			
		Range	Mean ± SD	Median (IQR)	*P* value^*c*^
HPV16 *n* = 133	+	17.1–40.4	29.5 ± 4.8	29.2 (6.8)	0.0002
	−	31.5–40.4	37.0 ± 3.5	37.8 (5.9)	
HPV18 *n* = 34	+	21.4–38.4	30.9 ± 5.2	31.2 (8.8)	0.0059
	−	37.2–38.3	37.6 ± 0.5	37.4 (0.7)	
12 pooled HPVs *n* = 334	+	16.2–40.0	29.9 ± 5.3	30.1 (8.6)	0.0000
	−	20.3–9.9	35.4 ± 4.9	37.6 (6.4)	

^*a*^ Ct, cycle threshold; HPV, human papillomavirus; HR, high risk; IQR, interquartile range; SD, standard deviation.

^*b*^ Includes only samples that had an HPV result by both tests and are positive by cobas for HPV.

^*c*^ Mann-Whitney test for median.

**TABLE 6:** Distribution of viral load for Anyplex according to HPV test results by cobas^*a*^^,^^*b*^

HR-HPV positivity by Anyplex	HPV test results by cobas	Viral load, Anyplex,^*c*^ *n* (%)			
		Low	Moderate	High	*P* value^*d*^
HPV16 *n* = 140	+	13 (61.9)	76 (91.6)	36 (100.0)	0.0000
	−	8 (38.1)	7 (8.4)	0	
HPV18 *n* = 35	+	6 (54.5)	16 (100.0)	8 (100.0)	0.0025
	−	5 (45.5)	0	0	
HPV31 *n* = 73	+	17 (81.0)	45 (97.8)	6 (100.0)	0.0161
	−	4 (19.0)	1 (2.2)	0	
HPV33 *n* = 26	+	3 (50.0)	15 (100.0)	5 (100.0)	0.0064
	−	3 (50.0)	0	0	
HPV35 *n* = 36	+	4 (100.0)	25 (100.0)	7 (100.0)	-
	−	0	0	0	
HPV39 *n* = 23	+	3 (60.0)	13 (92.9)	4 (100.0)	0.0636
	−	2 (40.0)	1 (7.1)	0	
HPV45 *n* = 32	+	8 (72.7)	17 (100.0)	4 (100.0)	0.0285
	−	3 (27.3)	0	0	
HPV51 *n* = 42	+	7 (53.9)	18 (90.0)	9 (100.0)	0.0041
	−	6 (46.1)	2 (10.0)	0	
HPV52 *n* = 47	+	17 (85.0)	22 (95.7)	4 (100.0)	0.1729
	−	3 (15.0)	1 (4.3)	0	
HPV56 *n* = 33	+	12 (85.7)	12 (85.7)	5 (100.0)	0.4935
	−	2 (14.3)	2 (14.3)	0	
HPV58 *n* = 49	+	8 (88.9)	20 (83.3)	16 (100.0)	0.2477
	−	1 (11.1)	4 (16.7)	0	
HPV59 *n* = 44	+	12 (92.3)	17 (89.5)	11 (91.7)	0.9495
	−	1 (7.7)	2 (10.5)	1 (8.3)	
HPV66 *n* = 48	+	13 (92.9)	21 (91.3)	10 (90.9)	0.8559
	−	1 (7.1)	2 (8.7)	1 (9.1)	
HPV68 *n* = 31	+	9 (64.3)	8 (57.1)	2 (66.7)	0.8836
	−	5 (35.7)	6 (42.9)	1 (33.3)	

^*a*^ HR, high-risk; HPV, human papillomavirus.

^*b*^ Includes only samples that had an HPV result by both tests and are positive by Anyplex for a given HPV. HPV test results by cobas for the 12 HR-HPVs, other than HPVs 16 and 18, represent a pooled result.

^*c*^ Categorized based on cycle threshold values as high (<31.0), moderate (31.0–40.0), and low (≥41.0).

^*d*^ Cochran-Armitage test for trend.

## DISCUSSION

Overall, Anyplex was slightly more likely than cobas to test positive for HPV16 (14.9% vs 13.4%), HPV18 (3.7% vs 3.6%), pooled HPVs (37.9% vs 34.3%), and any HPV (50.0% vs 45.3%). Our findings support previous reports of the consistently high agreement observed for HPV detection between cobas, currently used in clinical practice in many countries, and Anyplex (14–16). In a study among Korean women aged 23–81 years, the level of agreement between the two assays was 99.5% (kappa = 0.98) for HPV16, 99.8% (kappa = 0.96) for HPV18, and 98.8% (kappa = 0.97) for the pooled HPVs using 400 cervical samples (290 negative for intraepithelial lesions or malignancies, 40 with atypical squamous cells of undetermined significance, 22 with low-grade squamous intraepithelial lesions, 42 with high-grade squamous intraepithelial lesions, and 6 with atypical glandular cell samples) (14). In another study based on cervical samples from 403 Australian women (aged 30 years on average) undergoing management of high-grade cytological abnormalities (68 women with histologically confirmed low-grade abnormalities ≤ cervical intraepithelial neoplasia grade 1, cervical intraepithelial neoplasia grade 1 [CIN1]; 336 with histologically confirmed high-grade abnormalities ≥ CIN2), the overall agreement and kappa value for HPV detection by cobas and Anyplex were 96.0% and 0.87, respectively (15). The two assays were also concordant for HPV detection using 1,300 cervical specimens from women aged 25–64 years who participated in the organized cervical cancer screening program in Slovenia; the level of agreement was 99.5% (kappa = 0.96) for HPV16, 99.7% (kappa = 0.94) for HPV18, and 98.0% (kappa = 0.89) for the pooled HPV study (16).

To the best of our knowledge, only one study specifically examined the cobas pooled results for the 12 HPVs with individual positivity to these genotypes by the Anyplex II HPV28 assay, which differentiates between 19 HR and 9 low-risk HPVs (17). This study identified nine discordant samples: five (with normal histology) were negative by cobas but positive by Anyplex II HPV28 assay (three specimens HPV68+, one HPV16+, and one HPV59/69+) and four (two CIN1, one CIN2, one CIN3) were positive by both assays but for different HPV genotypes (17). In particular, cobas may underestimate the prevalence of HPV68 (a genotype considered as probably carcinogenic for cervical cancer) and has been found to have lower sensitivity for the HPV68a than HPV68b subtype (18). Our findings reinforce the increasing interest in triage strategies that incorporate genotype-specific risk stratification (19), rather than treating all high-risk types equally. For example, HPVs 31, 33, 45, 52, and 58 are associated with intermediate or variable risks for CIN3+, underscoring the need for more tailored clinical management approaches in future screening programs (20).

A few methodological complexities need to be discussed. First, study participants represent a mix of a screening and referral population, and hence the assays’ performance was not evaluated in a primary screening setting. The lack of histological verification in all women and differential disease sampling and ascertainment by country did not allow us to compare the sensitivity and specificity for disease detection between cobas and Anyplex. Second, although cobas and Anyplex testing were performed on two cervicovaginal samples collected simultaneously, which minimizes variability, minor differences in viral load distribution between samples could still account for some discordant results. Moreover, differences in DNA extraction methods and the time between sample collection and testing for each assay may have introduced further sources of variability. Third, we did not have specific information on the DNA region within the L1 region amplified and the primers used in the cobas assay. Although Anyplex is commonly described as targeting the L1 region (21, 22), recent evidence from a similar test developed by the same company (Allplex HPV28) suggests that amplification may also involve sequences from the E6/E7 regions (23). This uncertainty limits our ability to examine whether the differences observed between the two tests could be explained by differences in PCR techniques or the primers used. The tests may have different performances, depending on the specific HPV types, as was observed for HPV68.

In conclusion, detection of HR-HPV types by cobas and Anyplex was comparable. The added advantage of Anyplex lies in its further potential ability for follow-up testing and patient management by providing, beyond HPV16 and HPV18 positivity, individual genotyping information, which may be considered in future algorithms for the management of women with HR-HPV-positive Pap-negative test results, especially in populations with high vaccine coverage and considering HPV type replacement (24). Concurrent distinction of the 14 HR-HPV genotypes would be important to assess the impact of HPV vaccination on the distribution of HPV genotypes (25).

## Data Availability

The data will be deposited in an online research repository and can be made available upon request. Access is subject to prior approval from the data custodian(s) in each participating country, as the dataset is part of the ELEVATE consortium and includes sensitive clinical and demographic information. Due to ethical and legal constraints, public release is not permitted; however, qualified researchers may contact the corresponding author to request access, which will be evaluated by the consortium’s data governance committee.

## SUPPLEMENTAL MATERIAL

Table S1 jcm.00200-25-s0001.docx Cross tabulation [n (%)] between positivity to individual HR-HPV types by Anyplex, alone or in combination, and pooled HPV test results by cobas for the 12 other HR-HPV types.

ASM does not own the copyrights to Supplemental Material that may be linked to, or accessed through, an article. The authors have granted ASM a non-exclusive, world-wide license to publish the Supplemental Material files. Please contact the corresponding author directly for reuse.
