# Supplementary material for: Comparative performance of cobas 4800 HPV Test and Anyplex II HPV HR for high-risk human papillomavirus detection
Source: J Clin Microbiol. 2025 Jul 9;63(8):e00200-25. doi: 10.1128/jcm.00200-25 (PMC12345211; doi:10.1128/jcm.00200-25)
Supplement: Table S1 — Cross tabulation [n (%)] between positivity to individual HR-HPV types by Anyplex, alone or in combination, and pooled HPV test results by cobas for the 12 other HR-HPV types. [file jcm.00200-25-s0001.docx]

**Comparative performance of cobas 4800 HPV test and Anyplex II HPV HR for high-risk human papillomavirus detection**

Luani R. Godoy,^a,b,*^ Mariam El-Zein,^a*^ Elizaveta Padalko,^c^ Bo Verberckmoes,^d^ Bodine Van Eenooghe,^d^ Heleen Vermandere,^d^ Sónia Dias,^e^ Ana Gama,^e^ Bernardo Vega Crespo,^f^ Vivian Alejandra Neira,^g^ Eduardo L. Franco,^a^ Adhemar Longatto-Filho,^b,h,i,j^ for the ELEVATE Study Group^k^

^a^ Division of Cancer Epidemiology, McGill University, Montréal, Quebec, Canada

^b^ Molecular Oncology Research Center, Barretos Cancer Hospital, Barretos, São Paulo, Brazil

^c^ Department of Diagnostic Sciences, Faculty of Medicine and Health Sciences, Ghent University, Ghent, Belgium.

^d^ International Centre for Reproductive Health, Department of Public Health and Primary Care, Faculty of Medicine and Health Sciences, Ghent University, Ghent, Belgium

^e^ NOVA National School of Public Health, Public Health Research Centre, Comprehensive Health Research Center (CHRC), LA-REAL, NOVA University Lisbon, Lisbon, Portugal.

^f^ Facultad de Ciencias Médicas, Universidad de Cuenca, 010203, Cuenca, Ecuador.

^g^ Department of Biosciences, Faculty of Chemical Sciences, University of Cuenca. Cuenca, Ecuador^.^

^h^Life and Health Sciences Research Institute (ICVS), University of Minho, Campus de Gualtar, 4710-057 Braga, Portugal

^i^ICVS/3B’s—PT Government Associate Laboratory, 4710-057 Braga, Portugal

^j^Laboratory of Medical Investigation (LIM14), Faculty of Medicine, São Paulo State University, São Paulo 01049-010, Brazil

^k^ Yasmin M. Guimarães, Tauana C. Dias, Rui Manuel Reis (Barretos Hospital, Brazil), Olivier Degommme, Piet Cools, Marie Hendrickx (Ghent University, Belgium), José Ortiz Segarra (University of Cuenca. Ecuador).

**Supplementary Table 1.** Cross tabulation [n (%)] between positivity to individual HR-HPV types by Anyplex, alone or in combination, and pooled HPV test results by Cobas for the 12 other HR-HPV types

| **HPV positivity by Anyplex** | | **Cobas 12 pooled HPVs** | |
| --- | --- | --- | --- |
|  |  | **+** | **-** |
| **Single infections**^a^ | HPV31 | 34 (94.4) | 2 (5.6) |
|  | HPV33 | 13 (86.7) | 2 (13.3) |
|  | HPV35 | 16 (100) | 0 |
|  | HPV39 | 8 (80.0) | 2 (20) |
|  | HPV45 | 15 (83.3) | 3 (16.7) |
|  | HPV51 | 13 (81.2) | 3 (18.8) |
|  | HPV52 | 19 (82.6) | 4 (17.4) |
|  | HPV56 | 4 (66.7) | 2 (33.3) |
|  | HPV58 | 22 (84.6) | 4 (15.4) |
|  | HPV59 | 20 (87.0) | 3 (13.0) |
|  | HPV66 | 13 (92.9) | 1 (7.1) |
|  | HPV68 | 6 (35.3) | 11 (64.7) |
| **Multiple infections**^b^ | HPVs 16 and 31 | 4 (100) | 0 |
|  | HPVs 16 and 33 | 1 (50.0) | 1 (50.0) |
|  | HPVs 16 and 35 | 1 (100) | 0 |
|  | HPVs 16 and 39 | 1 (100) | 0 |
|  | HPVs 16 and 45 | 2 (100) | 0 |
|  | HPVs 16 and 51 | 1 (33.3) | 2 (66.7) |
|  | HPVs 16 and 52 | 1 (100) | 0 |
|  | HPVs 16 and 56 | 2 (100) | 0 |
|  | HPVs 16 and 58 | 1 (100) | 0 |
|  | HPVs 16 and 59 | 4 (100) | 0 |
|  | HPVs 16 and 66 | 2 (50) | 2 (50.0) |
|  | HPVs 16 and 68 | 1 (100) | 0 |
|  | HPVs 18 and 31 | 1 (100) | 0 |
|  | HPVs 18 and 39 | 0 | 1 (100) |
|  | HPVs 18 and 51 | 0 | 1 (100) |
|  | HPVs 18 and 52 | 1 (100) | 0 |
|  | HPVs 18 and 56 | 1 (100) | 0 |
|  | HPVs 18 and 66 | 1 (100) | 0 |
|  | HPVs 18 and 68 | 0 | 1 (100) |
|  | HPVs 31 and 33 | 1 (100) | 0 |
|  | HPVs 31 and 35 | 1 (100) | 0 |
|  | HPVs 31 and 45 | 2 (100) | 0 |
|  | HPVs 31 and 51 | 1 (100) | 0 |
|  | HPVs 31 and 52 | 1 (100) | 0 |
|  | HPVs 31 and 56 | 1 (50.0) | 1 (50.0) |
|  | HPVs 31 and 58 | 1 (100) | 0 |
|  | HPVs 31 and 59 | 2 (100) | 0 |
|  | HPVs 31 and 66 | 4 (100) | 0 |
|  | HPVs 31 and 68 | 1 (100) | 0 |
|  | HPVs 33 and 58 | 1 (100) | 0 |
|  | HPVs 33 and 59 | 1 (100) | 0 |
|  | HPVs 33 and 66 | 2 (100) | 0 |
|  | HPVs 35 and 45 | 1 (100) | 0 |
|  | HPVs 35 and 51 | 1 (100) | 0 |
|  | HPVs 35 and 52 | 1 (100) | 0 |
|  | HPVs 35 and 56 | 4 (100) | 0 |
|  | HPVs 35 and 58 | 1 (100) | 0 |
|  | HPVs 35 and 59 | 1 (100) | 0 |
|  | HPVs 35 and 66 | 1 (100) | 0 |
|  | HPVs 39 and 45 | 1 (100) | 0 |
|  | HPVs 39 and 51 | 2 (100) | 0 |
|  | HPVs 39 and 58 | 1 (100) | 0 |
|  | HPVs 39 and 59 | 1 (100) | 0 |
|  | HPVs 39 and 66 | 1 (100) | 0 |
|  | HPVs 45 and 56 | 1 (100) | 0 |
|  | HPVs 45 and 66 | 1 (100) | 0 |
|  | HPVs 51 and 52 | 1 (100) | 0 |
|  | HPVs 51 and 56 | 1 (100) | 0 |
|  | HPVs 51 and 58 | 1 (100) | 0 |
|  | HPVs 51 and 66 | 2 (100) | 0 |
|  | HPVs 51 and 68 | 2 (100) | 0 |
|  | HPVs 52 and 58 | 4 (100) | 0 |
|  | HPVs 52 and 59 | 2 (100) | 0 |
|  | HPVs 52 and 66 | 1 (100) | 0 |
|  | HPVs 56 and 58 | 1 (100) | 0 |
|  | HPVs 56 and 59 | 1 (100) | 0 |
|  | HPVs 56 and 66 | 2 (100) | 0 |
|  | HPVs 58 and 66 | 1 (100) | 0 |
|  | HPVs 59 and 66 | 1 (100) | 0 |
|  | HPVs 59 and 68 | 2 (100) | 0 |
|  | HPVs 66 and 68 | 1 (100) | 0 |
|  | HPVs 16, 18, and 51 | 1 (100) | 0 |
|  | HPVs 16, 31, and 35 | 1 (100) | 0 |
|  | HPVs 16, 31, and 51 | 1 (100) | 0 |
|  | HPVs 16, 31, and 52 | 1 (100) | 0 |
|  | HPVs 16, 31, and 59 | 1 (100) | 0 |
|  | HPVs 16, 33, and 66 | 1 (100) | 0 |
|  | HPVs 16, 39, and 68 | 1 (100) | 0 |
|  | HPVs 16, 45, and 58 | 2 (100) | 0 |
|  | HPVs 16, 51, and 58 | 1 (100) | 0 |
|  | HPVs 16, 58, and 66 | 0 | 1 (100) |
|  | HPVs 16, 58, and 68 | 1 (100) | 0 |
|  | HPVs 18, 33, and 35 | 1 (100) | 0 |
|  | HPVs 18, 39, and 52 | 1 (100) | 0 |
|  | HPVs 18, 52, and 58 | 1 (100) | 0 |
|  | HPVs 31, 39, and 51 | 1 (100) | 0 |
|  | HPVs 31, 51, and 59 | 0 | 1 (100) |
|  | HPVs 31, 52, and 56 | 1 (100) | 0 |
|  | HPVs 31, 56, and 66 | 1 (100) | 0 |
|  | HPVs 31, 56, and 68 | 1 (100) | 0 |
|  | HPVs 31, 58, and 66 | 2 (100) | 0 |
|  | HPVs 31, 59, and 68 | 1 (100) | 0 |
|  | HPVs 33, 35, and 66 | 1 (100) | 0 |
|  | HPVs 33, 52, and 66 | 1 (100) | 0 |
|  | HPVs 35, 52, and 66 | 1 (100) | 0 |
|  | HPVs 35, 56, and 68 | 1 (100) | 0 |
|  | HPVs 45, 52, and 56 | 2 (100) | 0 |
|  | HPVs 16, 18, 35, and 52 | 1 (100) | 0 |
|  | HPVs 16, 18, 56, and 66 | 1 (100) | 0 |
|  | HPVs 16, 31, 51, and 56 | 0 | 1 (100) |
|  | HPVs 16, 31, 51, and 59 | 1 (100) | 0 |
|  | HPVs 16, 31, 56, and 58 | 1 (100) | 0 |
|  | HPVs 16, 39, 45, and 66 | 1 (100) | 0 |
|  | HPVs 18, 35, 51, and 52 | 1 (100) | 0 |
|  | HPVs 18, 39, 58, and 59 | 1 (100) | 0 |
|  | HPVs 31, 51, 52, and 56 | 1 (100) | 0 |
|  | HPVs 35, 45, 51, and 59 | 1 (100) | 0 |
|  | HPVs 51, 56, 58, and 66 | 1 (100) | 0 |
|  | HPVs 52, 56, 66, and 68 | 1 (100) | 0 |

HR, high-risk; HPV, human papillomavirus

^a^ Includes samples that had an HPV result by both tests and are positive for only one HPV type by Anyplex.

^b^ Includes samples that had an HPV result by both tests and are positive for ≥2 HPV types by Anyplex.
